# Patient safety in wound care: experiences of healthcare professionals in home healthcare settings

**DOI:** 10.1080/17482631.2026.2682039

**Published:** 2026-06-02

**Authors:** Victoria Sandholm, Ingrid Larsson, Petra Svedberg, Anna Gyberg

**Affiliations:** a School of Health and Welfare, Halmstad University, Halmstad, Sweden

**Keywords:** Patient safety, wound care, healthcare professionals, qualitative methods, home healthcare, municipal care

## Abstract

**Background:**

Home healthcare includes many safety challenges in wound care due to the unique and varied care conditions. In this specific context, there is a paucity of knowledge regarding how healthcare professionals ensure safety in wound care management. This study aimed to explore how patient safety risks are experienced and managed in wound care by healthcare professionals in Swedish home healthcare settings.

**Design:**

The study was based on a qualitative, explorative research design with an inductive approach.

**Method:**

Individual interviews were conducted with 16 healthcare professionals with wound care experience in home healthcare services. The data was analysed based on qualitative content analysis.

**Results:**

Two overarching themes and six subthemes were identified as influencing patient safety, presenting challenges that healthcare professionals were required to manage on a daily basis: “Working blindfolded” reflected challenges with the many healthcare professionals involved in wound care. Challenges also comprised fragmented communication pathways and varying knowledge levels, which at times required them to work without the full information or knowledge needed. “Working with hands tied” concerned how registered nurses were obliged to work within specific frameworks with limited possibilities to act. Preventative work was curtailed, reaching agreements with other members of the wound care team that proved challenging in a diverse and dynamic environment.

**Conclusion:**

The findings predominantly indicated challenges related to organisational preconditions that could jeopardise patient safety. In certain instances, the professionals were unable to resolve these issues, which resulted in an imminent risk of adverse events and prolonged wound healing.

## 1. Introduction

Wounds constitute a major health burden, with significant consequences for individuals and healthcare systems. Beyond having a considerable impact on patients’ quality of life, hard-to-heal wounds generate economic costs for healthcare services through intensive treatments and extended care (Olsson et al., 2019). A previous literature review demonstrated a pooled global prevalence of 2.21 per 1000 population for hard-to-heal wounds with mixed aetiologies (Martinengo et al., 2019). Some wounds are caused by surgical procedures or trauma, while others develop secondary to prolonged pressure or long-term conditions such as diabetes (Lindholm & Searle, 2016). Hard-to-heal wounds are associated with higher age and comorbidities, and the incidence of hard-to-heal wounds can therefore be expected to increase with an ageing population (Kielo et al., 2020; Lindholm & Searle, 2016; Olsson et al., 2019). Ensuring safe patient care is a major challenge in home healthcare, where wound healing may be delayed or fail due to comorbidities and challenges related to the home healthcare setting (Alvarez-Irusta et al., 2022). Maintaining patient safety and avoiding adverse events during wound care in home healthcare demands a wide range of knowledge and skills among healthcare professionals (Kielo et al., 2020). However, there is a paucity of research concerning how healthcare professionals ensure safe wound care. This study focuses on wound care from a patient safety perspective, with particular emphasis on how healthcare professionals experience and manage risks while maintaining patient safety in home healthcare settings.

In this context, adverse events such as healthcare-associated infections and pressure ulcers are prevalent (Pejner & Kihlgren, 2019; Schildmeijer et al., 2018). These types of adverse events can result from suboptimal wound care management (Alvarez-Irusta et al., 2022). Patient safety, defined as the work done to minimize the risk of patient harm or the impact of patient harm, requires organized activities that “*create cultures, processes, procedures, behaviours, technologies and environments*” that support such work (World Health Organisation, 2021, p. V.). However, actualizing such a framework has proven to involve several challenges. For example, studies have shown that wound care is fragmented, with multiple actors in the healthcare system working in isolation (Alvarez-Irusta et al., 2022; Schildmeijer et al., 2018). This fragmentation was recognized as a patient safety risk and associated with poor health outcomes, stemming from discontinuities in care. Another challenge is the limited access to registered nurses and physicians, which has negative effects on patient safety (Schildmeijer et al., 2018). The delegation of nursing tasks is common in elder care; for example, 68% of nursing interventions are performed by unlicensed staff (Norell et al., 2013). Consequently, within municipal home healthcare settings, tasks such as wound care are often carried out by staff with varying levels of competence (Ekstedt et al., 2022). This lack of competence is known to increase the incidence of adverse events (Lekman et al., 2023). These challenges place significant demands on effective communication between care providers—a critical factor that has been shown to impact patient safety (Lindh & Sahlqvist, 2012). Not least, the home environment is fundamentally different from other care settings and usually not adapted to care. Consequently, challenges arise due to a lack of fit between available resources and care demands, along with an environment that is not supportive of hygienic wound care (Ekstedt et al., 2022). To summarize, patient safety in home healthcare settings relies on appropriate competence and skills, and a well-functioning organization and communication.

### 1.1. Perspectives on patient safety

In this study, we conceptualize patient safety as both action and non-action, and as an ongoing negotiation among all the actors involved in ensuring the patient’s safety, including the patient, relatives, and healthcare professionals (Hor et al., 2013; Iedema et al., 2012; Sahlsten et al., 2007). In wound care, patients assume an important role in managing wounds at home, as they have the capacity to positively influence outcomes (Huang et al., 2023). Consequently, they also constitute an integral part of patient safety work. Although patients play a key role in patient safety because of their ability to identify safety risks and, to a great extent, address and resolve them themselves (Gyberg et al., 2024), our focus is on the healthcare professionals. They ultimately hold responsibility for patient safety within the context of another person’s home, a setting that has been shown to be particularly challenging and to require adequate communication and collaborative structures in cross-disciplinary work in primary and municipal care (Silverglow et al., 2022). Patient safety in home healthcare services largely depends on the staff working closest to the patient, as they are responsible for managing safety in their everyday practice (Ekstedt et al., 2022). In recent decades, the conventional view of patient safety has evolved from a primary focus on individual human errors to a broader perspective that also encompasses organizational and systemic factors (Cook, 2013). This shift highlights the need to understand the range of factors that can contribute to patient safety risks in home healthcare. The majority of research on patient safety in home healthcare has concentrated on the types of adverse events and safety risks, primarily emphasizing what went wrong and adopting a retrospective problem-solving approach (Sahlqvist & Härenstam, 2024). However, to create safe healthcare and to foresee and prevent adverse events, there is also a need to understand why things go right (Hollnagel, 2017). The everyday performance of healthcare professionals succeeds more often than it fails, as they constantly adapt their work, depending on varying conditions, for example, when communication, routines or processes do not flow as expected (Braithwaite et al., 2015). Cook et al. (2000) imply that these discontinuities, or gaps, can pose a threat to patient safety. However, healthcare professionals anticipate, identify, and manage such gaps – both consciously and unconsciously – in their daily work. Thus, in practice, Cook et al. (2000) mean that gaps rarely result in overt failures, as their consequences are mitigated by those working closest to the patients. This highlights the critical role of healthcare professionals at the sharp end of care in maintaining patient safety. Understanding healthcare professionals’ ability to detect and bridge gaps is therefore essential to improving patient safety (Cook et al., 2000).

### 1.2. Problem statement

The majority of research on patient safety in home healthcare settings has focused on identifying existing patient safety risks (Shahrestanaki et al., 2023; Silverglow et al., 2023). However, little is known about how healthcare professionals actively manage these risks. This is especially relevant in the context of wound care in the patients’ home, a setting that previous research has demonstrated to be challenging (Ekstedt et al., 2022; Lekman et al., 2023). To improve patient safety in home healthcare, there is a need to understand healthcare professionals’ everyday practice and explore how they manage patient safety risks in varying situations. Given the contextual nature of patient safety, an in-depth examination of wound care at the point of care may yield new insights that can inform advancements in patient safety practices. Therefore, the aim of this study was to explore how patient safety risks are experienced and managed in wound care by healthcare professionals in Swedish home healthcare settings.

## 2. Materials and methods

### 2.1. Design

The study employed a qualitative, exploratory design with an inductive approach to explore experiences of wound care from a patient safety perspective in home healthcare. This design was considered appropriate for gaining an in-depth understanding of participants’ experiences of a relatively underexplored phenomenon. The inductive approach allowed patterns and categories to emerge from the data without being guided by predetermined assumptions. The study is reported in accordance with the Consolidated Criteria for Reporting Qualitative Research (COREQ) (Tong et al., 2007).

### 2.2. Setting and participants

The study included two relatively small municipalities in southern Sweden, with respective populations of approximately 10,000 and 25,000. The municipalities were located in rural areas with low population density and without any large cities. The responsibility of providing home healthcare in Sweden is decentralized to the municipal level, as opposed to physicians in primary care, who are employed at the regional level (Pejner & Kihlgren, 2019). Primary care physicians are, however, frequently involved through consultations, for example, in conducting medical assessments of wounds. In municipal healthcare, nurses and nursing assistants play a key role in wound care. Therefore, the data sampling was purposive in terms of recruiting nurses and nursing assistants with wound care experience. To ensure diversity, a variation in gender and age was also considered. From February to October 2023, invitations to participate were distributed to the first-line managers from the two municipalities, who forwarded the request to all relevant staff members who met the inclusion criteria. In total, 32 nurses and 14 nursing assistants were invited to participate in the study, of which a total of 16 agreed to participate. The participants in the study comprised nine registered nurses, four district nurses and three nursing assistants who worked in home healthcare or special care homes and had wound care experience varying from 2 months to 27 years (see Table I).

**Table I.** Characteristics of the participants.

Total number of participants	16
Municipality A	13
Municipality B	3
Male	1
Female	15
**Mean age (range)**	42.7 (24–56)
**Professions**	
Nursing assistant	3
Registered nurse	9
District nurse	4
**Years in the workplace, median (range)**	5 (<1–23)
**Experience in wound care, median (range)**	10 (<1–27)

In Sweden, registered nurses and nursing assistants working in home healthcare are employed under different legislation. Nursing assistants responsible for basic nursing care are governed by the Social Services Act (SoL) (2001:453), whereas registered nurses are governed by the Swedish Health and Medical Service Act (HSL) (2017:30). Consequently, nursing assistants and registered nurses work in separate patient records. However, some nursing assistants are employed under the same legislation as registered nurses (HSL) and are here referred to as nursing assistants (HSL), in contrast to those employed under the Social Services Act, referred to as nursing assistants (SoL). This gives them access to the same patient record as the registered nurses and allows them to take on more advanced tasks assigned directly by nurses, such as wound dressing. Consequently, nursing assistants (HSL) have access to more information than nursing assistants (SoL).

### 2.3. Data collection

Individual interviews took place in a face-to-face setting at the healthcare organization's premises, with only the interviewer and interviewee present. The interviews were conducted by two experienced researchers whose combined expertise included interview techniques, healthcare organization, and nursing. All participants gave verbal and written consent to participate in the study. An interview guide was used to ensure consistency across the interviews. A pilot interview was conducted to test the questions. As no changes were made to the interview guide, the interview was included in the analysis. The interview guide was structured around key themes and supported by guiding questions. While the questions were primarily designed to align with the study aim, they were allowed to be adapted to the context of issues raised by participants, given that patient safety is situation-specific and can be experienced from multiple perspectives. In practice, all interviews began with an open question asking the participant to describe their work and tasks, before moving on to more targeted questions about wound care and patient safety. Examples of these questions were “what does patient safety mean to you?”, “what opportunities currently exist to ensure patient safety?” and “what improvements are needed to enhance patient safety in wound management?”. Follow-up questions were frequently used to gain deeper knowledge into the challenges, possibilities, and potential improvements to patient safety work. For example, the follow-up questions “does this affect patient safety and, if so, how?” and “how is/was this risk managed” were asked. Overall, the topic of patient safety centred on the participants’ experiences of patient safety risks and on how their patient safety work was carried out, that is, how such risks were managed. The audio-recorded interviews lasted between 49 and 116 minutes (mean = 71 minutes) and were transcribed verbatim in Swedish. The analysis was performed using the original Swedish transcripts. Only quotations selected for illustrative purposes were translated into English.

### 2.4. Data analysis

To capture the participants' experiences of patient safety risks and management, qualitative content analysis was used to analyze the data, in accordance with Graneheim and Lundman (2004); Graneheim et al. (2017). The method enabled the identification of patterns through a process of interpretation and abstraction of data. The interviews were initially thoroughly read to gain an overall understanding. This was followed by a decontextualisation of the text into smaller meaning units, consisting of sentences or paragraphs related to the research aim. The meaning units were then coded, which reflected our interpretation of the underlying meaning, and different levels of themes were generated through a process of abstraction and interpretation. As the data was rich and expressive, the analysis was performed by moving directly from codes to sub-themes and themes (Graneheim & Lundman, 2004; Graneheim et al., 2017). Thus, the analysis included latent interpretations that captured underlying patterns throughout the data (Lindgren et al., 2020). The confirmability of the study was enhanced by involving all authors in the analysis, each contributing from their respective roles. Specifically, one researcher conducted the initial coding, another validated the coding and challenged interpretive decisions, while the third and fourth researchers provided reflective input throughout the analytical process. Additionally, the consistent use of quotations to trace the basis of the interpretations further enhanced the transparency of the analysis. The analysis generated 285 meaning units, six sub-themes, and two main themes. These numbers are presented to illustrate the scope of the analytic process, while theme development was based on interpretive meaning and patterns in the data rather than on frequency. An example of the analysis process is shown in Table II.

**Table II.** Illustration of the analysis process.

Meaning unit	Condensed meaning unit	Code	Subtheme	Theme
“Unfortunately, there has been a period with a lot of people coming and going, so to speak, which I don't think anyone really wants. Instead, we want continuity so that we can follow both the person and changes in their well-being and...” (Participant 15)	Many people in circulation and lack of continuity among patients makes it difficult to monitor patients' well-being.	Lack of continuity in patient care creates difficulties in follow-up	Figuring out what the other hand is doing	
“I don’t have access to that (documentation system). You have to wait for them to notify you by phone, and very often you get a message saying "that patient was ill", and I see that the next day. I think ‘But why didn't you call about this?’ Someone just wrote a message, and sometimes they only wrote it to me. And I mean, what if I'm not there for three days? So, no... the communication is difficult.” (Participant 8)	Because of legal restrictions, important information is missed. Calls are sometimes not made, and messages can remain unread for several days.	Access to different types of information is regulated by different laws	Compensating for fragmented documentation	*Working blindfolded*
“What makes you feel the worst is to see that the quality of care out there, both in special care homes but also in home care, is increasingly lacking, with employees who do not have the knowledge or the readiness or the will, or whatever it is. That the care is not as good as it has in fact been before.” (Participant 5)	The quality of care is increasingly lacking, with staff who do not have sufficient knowledge, readiness or willingness.	Lack of understanding lowers the quality of care.	Acting without sufficient knowledge	
“For instance, no risk assessments have been conducted. There's been no effort to work preventively and tackle issues like falls, pressure, and nutrition. The problems have already cropped up. And then you have to take it from there. Unfortunately, that's not the approach we're taking today.” (Participant 24)	Risk assessments aren't being done, and there's been no effort to tackle issues like falls, pressure, and nutrition in a preventative way.	Lack of preventive work	Spending time putting out fires	
“We often get the comment (from the doctor) ‘But you know this better than me.’ Well, no, we don’t—that’s why we’re asking. But then he just refers us on to the district nurse in primary care.” (Participant 4)	When nurses request the doctor’s support, the doctor refer back to the nurses as having greater expertise.	Access to primary care support is limited.	Spending time convincing others	*Working with hands tied*
“It's not easy to work in people's homes. You go to people's homes and they have it their way, and you want to wash in a clean environment with soap and water, but you can't always use the bathroom, and it may not be clean. You can't really do much about it. It's their choice how they live, but it's difficult to work. It's not like a hospital or in primary care where you have a bed.” (Participant 11)	With limited access to clean facilities this challenge the safety of wound care in home settings.	Inadequate conditions in home settings	Making the most of existing resources	

### 2.5. Qualitative rigour

Qualitative research depends on researcher interpretations, and thus the researchers' reflexivity in ensuring the quality and trustworthiness of the research process (Alvesson & Sköldberg, 2017; Olmos-Vega et al., 2022). Several reflexivity practices were used to develop a rigorous study. Researchers with diverse pre-understandings, encompassing both clinical and theoretical perspectives on wound care and patient safety, were involved in the analysis to enhance trustworthiness and reduce potential bias stemming from individual perspectives.

The researchers returned to the data throughout the process of the analysis to ensure that the interpretations were true to the data. Consensus was achieved throughout the analysis regarding the development of themes in relation to the data (Graneheim & Lundman, 2004). The steps of the analysis were carefully presented to ensure trustworthiness and transparency, with quotations used to clarify the basis of the interpretations. Furthermore, quotations were selected to represent the variety of roles within the healthcare organization, to include a range of perspectives, and to make the interpretations transparent to the reader (Elo et al., 2014).

### 2.6. Ethical considerations

The study was approved by the Swedish Ethical Review Authority (no. 2022-05837-01 and 2023-02581-02). All participants received verbal and written information about the study and gave informed consent prior to participation. Participation was voluntary, and participants were informed of their right to withdraw at any time without explanation. All participants’ confidentiality was protected, and quotations were presented in a way that ensures that the identification of individuals is not possible, in accordance with the Helsinki Declaration (World Medical Association, 2024). To maintain confidentiality, the names of individuals cited in the text have been fictitiously altered.

## 3. Findings

**Figure 1.**
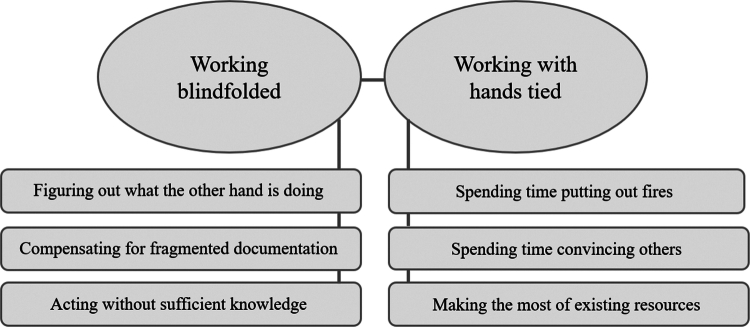
Themes and subthemes reflecting healthcare professionals’ experiences of how patient safety risks are experienced and managed in wound care in Swedish home healthcare.

Two themes and six subthemes were identified in the data (see Figure 1); “Working blindfolded” reflected the challenges of having several different healthcare professionals involved in wound care, which created discontinuity. It also reflected challenges involving fragmented communication pathways and varying knowledge levels, which forced the healthcare professionals to work without the full information or knowledge needed. “Working with hands tied” concerned how nurses were forced to work within specific frameworks, with limited scope to act. This led to a lack of optimization of the preventive work and made it hard to reach agreements with other members in the wound care team in a diverse and dynamic environment. Basically, “Working blindfolded” and “Working with hands tied” come down to the healthcare professionals’ limited preconditions to know how to promote safe wound healing and to act in a timely and appropriate manner. Together, these themes demonstrate that patient safety risks were predominantly associated with inadequate preconditions of home healthcare organizations. This was a concern among the participants, as it presented an imminent risk of prolonged wound healing, adverse events, and, consequently, patient suffering.

### 3.1. Working blindfolded

The first identified theme, “working blindfolded”, illustrated how healthcare professionals had to engage in wound management without sufficient knowledge or information. Discontinuity in patients' visits and wound assessments, combined with fragmented documentation, led to gaps in information exchange and patient safety risks. Additionally, the variation of education and training among staff members placed an extra burden on experienced staff to maintain safe wound care.

#### 3.1.1. Figuring out what the other hand is doing

A patient safety risk identified by the participants was the large number of healthcare professionals involved in wound care. The problem with several healthcare professionals, and thus the many “hands”, made it difficult to follow the treatment and healing of wounds, which could pose a risk for, e.g., infections or prolonged healing processes, owing to inconsistency in material choices and treatment.

*“Yes, it's the same with the wounds. It's not possible to keep track of it all. In fact, it's not even ten, it's... well, you know what they* (the patients) *say here, "I've had 80 different nurses... "* (…) *So if there are as many people touching a wound, it is clear that it will never be good.”* [participant 1]

It was also evident that the lack of continuity in patients' visits made it challenging to detect a potential deterioration of the patients’ health condition, which could result in adverse events. In addition, participants experienced that this discontinuation could contribute to a sense of insecurity and reluctance among patients to share information that potentially could require attention. To manage this problem, registered nurses frequently made efforts to reduce the number of healthcare professionals involved in the patients’ wound care. As it was difficult to achieve optimal continuity, the nurses were forced to rely heavily on other staff members to communicate identified abnormal signs, e.g., redness, which could indicate the beginning of a pressure ulcer. As in the following example, limiting the number of staff involved could be prioritized dependent on the type of wound:

*“It's a bit different if it's a bigger wound or one that's difficult to heal. We'll probably have the nurses change the wound dressing, maybe primarily the nurse in charge, so that one person can take care of it. Otherwise... we don't want too many people involved, because it's easier to determine whether the wound is healing or not.”* [participant 2]

Despite efforts to provide as much continuity as possible, the findings showed that it was a recurring patient safety challenge to organize and manage the work in terms of who should do what and in what way, without having too many people involved in wound care.

#### 3.1.2. Compensating for fragmented documentation

The findings showed that the wound care documentation was fragmented, i.e., that wound assessment, wound appearance, and the wound care plan were documented in different ways and in different places. This fragmented documentation poses a risk to patient safety for two main reasons. Registered nurses and nursing assistants operated under two distinct legislative frameworks, which created a barrier in the exchange of comprehensive information, as they had separate documentation systems. This resulted in access to information being largely dependent on verbal communication channels between team members to gain insight into the wound care plan and documentation. The nursing assistants (HSL), who had access to both documentation systems, were frequently mentioned as *“acting as a bridge between the different laws”* [participant 4] and hence as information carriers with their dual authority.

*“I have to rely quite a lot on others sharing information with me, and sometimes you think "Oh, why didn't you tell me this? You should have."* [participant 4]

The second reason for the fragmentation was that the documentation was done in different ways from one individual to another. Consequently, important information was sometimes not reported in sufficient detail, which posed a risk to safe wound healing. Some thought that these differences were due to variations in knowledge and interest. The different approaches were also a consequence of the conditions for documentation. For example, without a computer in the patient's home and with a busy schedule, documentation could be kept on sticky notes, pending entering the information into the medical record. This could lead to delays in sharing important information with others, which could create the risk of colleagues leaving out or duplicating tasks that had already been completed. Additionally, when information was documented after all daily visits, its accuracy could be influenced by memory loss, potentially compromising the next shift's ability to provide safe wound care. It was furthermore clear that words were not enough to provide a comprehensive understanding of wound appearance or to follow their healing. To solve this problem, photo documentation was adapted as a solution despite the absence of formalized procedures. By using photo documentation, the healthcare professionals were able to bridge communication gaps and maintain continuity in treatment, despite the inherent risks to patient privacy.

*“I think that writing just "had a wound on left lower leg that I dressed" doesn't give much information. I don't know the size or depth of the wound, whether it was oozing, whether it was healing, or whether it was becoming dead tissue. I don't know anything.”* [participant 14]

The issue of fragmented documentation resulted in the healthcare professionals becoming isolated knowledge holders regarding patients' wounds and treatments. Thus, patient safety was dependent on how each healthcare professional chose to document their observations and actions. The findings also highlighted that patient safety depended on informal methods, such as using photos to facilitate communication and monitor healing across different teams.

#### 3.1.3. Acting without sufficient knowledge

A prominent patient safety challenge was the variation in knowledge and education levels. Some care workers, who were responsible for providing daily personal care and assistance but were not involved in wound treatment, lacked formal training. Additionally, the educational opportunities that were offered to staff members internally started long after employment and did not feel comprehensive enough. The findings showed significant concerns about how a lack of training could negatively affect the quality of wound care, posing a risk to patient safety. The lack of understanding of wound progression and basic care could result in care workers not reporting, for example, red marks, which subsequently could minimize the opportunity to prevent pressure ulcers from developing. The participants associated the lack of sufficient knowledge with difficulties in applying a holistic perspective on the patient. As one participant expressed:

*“It seems that anyone can be brought in just to fill a spot, and this is also noticeable in the problem with pressure ulcers that has increased as a result. The basic care is left out, and then the pressure ulcers occur more frequently. I don't think we've had as many pressure ulcers as we have now.”* [participant 2]

Sufficient knowledge was regarded as essential for creating favourable conditions for wound prevention and healing. Furthermore, the lack of knowledge made inexperienced staff members dependent on more experienced nurses. Although it was desired that less experienced staff reach out to senior colleagues for help, these interruptions compromised the senior nurses' ability to prioritize effectively, creating additional work and delays to ongoing tasks.

*“I mean, sometimes you get a call from them saying you must come over right away because it's bleeding. So, you reschedule your day and head over. And then you find it's almost like you need a magnifying glass to see it.”* [participant 11]

One way to manage the knowledge variation was to assign tasks based on the level of expertise. Despite these efforts, it seemed that the knowledge gaps were a problem with no silver lining, and this had a negative effect on patient safety.

### 3.2. Working with hands tied

The second theme showed that the participants were working in situations that forced them to operate with “their hands tied”, meaning that they, in some situations, were not free to perform preventive initiatives for wound care. This was expressed in situations where they were required to adopt a problem-oriented perspective on nursing. Moreover, the participants were dependent on medical assessment and decisions made in primary care, which further limited their ability to act autonomously. This dependency on others was also observed in situations where the patient’s perceived needs did not align with the registered nurse’s assessment. Wound care was also dependent on the conditions and setting of each individual home, requiring the healthcare professionals to navigate diverse home conditions. Overall, the preconditions for registered nurses to influence patient safety work were sometimes beyond their control.

#### 3.2.1. Spending time putting out fires

Prominent throughout the findings was a problem-oriented mission, which hindered preventive work. Owing to staff shortages, time efficiency, and the distribution of tasks, participants expressed that they were hindered from engaging in preventive work, thereby missing the opportunity to participate in basic nursing care. This resulted in registered nurses spending time “putting out fires”, only being able to act when there was already a developed wound. Thus, opportunities to prevent adverse events such as pressure ulcers and wound infections were limited. The registered nurses expressed that small gestures, such as warming a patient's dinner while simultaneously observing their mobility, were not part of the registered nurse's duties. Hence, they missed the chance to identify changes in the overall behaviour and health of patients. Some registered nurses also expressed a culture in which they felt reluctant to intervene and control the care, due to risks of offending other staff members. One participant described the limited influence on the management of pressure ulcer prevention as follows:” I *can't intervene with that. But 'oh, there's a wound'. Then I come in.”* [participant 5]. Another participant expressed:

*“So, it's the firefighting; you have to deal with problems here and now instead of having a ready-made plan.”* [participant 16]

In managing patient safety, the findings showed that registered nurses worked behind the scenes, making efforts to express their recommendations and influence basic and preventive wound care. With insufficient time, due to staff shortages, the participants emphasized the importance of allocating tasks for wound treatment depending on the existing staff’s experience and the healing potential of the wounds. The excessively narrow and problem-oriented mission that was prominent through the findings appeared to diminish the holistic perspective on the patient. If they were hindered in operating to their full capacity as registered nurses, preventive measures were limited, and adverse events could occur.

#### 3.2.2. Spending time convincing others

The findings revealed challenges in achieving coordinated efforts among registered nurses, nursing assistants, general practitioners, and the patients. Thus, time was spent on convincing others, which implied the need for both the patient and other healthcare providers to pull together to be able to ensure safety for patients. As a first example, the participants described a balancing act between respecting the patient’s wishes and providing the most suitable wound treatment. Participants addressed that some patients were reluctant to have their wounds treated or be involved in wound treatment. In these situations, it was perceived as important that there were good relationships between the healthcare professionals and the patients. Thus, the participants made attempts to invest time in establishing trustful relationships, to ensure a collaboration in which the patient and the healthcare professionals pulled together. One way to build trust was by trying to have continuous patient visits with as few healthcare professionals involved as possible. It was also important to involve the patient in the wound care plan and self-care, as in the following quotation.

*“So, you start from the person you have in front of you, what they ... they should be involved. Because it's difficult to get going, i.e. to achieve any improvement, if you don't have the patient with you.”* [participant 14]

As a second example, the challenge of being dependent on others was also seen in situations where registered nurses needed medical support, e.g., diagnosis and decide on treatment strategies. The collaboration with primary care was sometimes expressed as complicated and inaccessible, as several participants experienced difficulties when turning to physicians for advice. For example, physicians appeared to expect registered nurses to have sufficient knowledge, sometimes even surpassing their own, which may explain why some participants expressed that some wounds were left undiagnosed and without a treatment plan. It was also stated that physicians sometimes prescribed treatments without being aware of the wound's appearance. Receiving no or insufficient medical support allows the registered nurses to assess wounds and decide on treatment based on their own knowledge and experience.

*“We have some good primary care physicians, absolutely. But unfortunately, when you ask them how to dress a wound, you very often get the answer “do what you usually do” … and that's the worst answer I can get”.* [participant 11]

These findings emphasize that registered nurses and nursing assistants can only ensure patient safety to a certain extent, as their work is dependent on sufficient collaboration. It appeared that wound healing could be impeded without appropriate medical support and without trusting relationships with patients. Thus, these factors could potentially compromise the safety and effectiveness of wound care.

#### 3.2.3. Making the most of existing resources

The findings showed several patient safety challenges relating to wound care in the home setting that were linked to making the most of existing resources. The patients’ homes were not adequately equipped with the necessary materials. Limited access to resources, combined with long distances for obtaining equipment in rural areas, was perceived to contribute to delayed wound healing. The participants expressed that they only brought the supplies needed for each wound dressing, leaving them unprepared for unexpected situations. Another consequence was that important measurements and assessments were sometimes left out, because they could not bring all the necessary equipment to the patient’s home.

*“We have half an hour from the office* [to the patients´ home]*, and then it becomes a slightly different dilemma if you need to pick something up.”* [participant 13]

Another challenge was the prerequisites regarding sanitation and lighting. Poor sanitation hindered wound cleanliness and negatively impacted the healing process, while inadequate lighting and suboptimal working positions further complicated effective wound treatment. Ensuring patient safety often necessitated improvisation and resourcefulness, such as utilizing available materials to dress acute wounds when standard supplies were lacking. Some expressed that they made sure to bring material and leave it for the next colleague when visiting a patient.

*“Yes, you just do your part anyway to the best of your ability. You might pick up a kitchen chair as a wound care setup, so you just have to improvise.”* [participant 14]

Ensuring patient safety was closely tied to the contextual conditions of each patient, and was complicated by the participants’ limited capacity to foresee changes in the patients’ situations. This posed a particular risk in situations where patients required hospital care but were reluctant to go to the hospital. Prominent throughout the findings was the fact that the healthcare professionals had to adapt to the conditions of each individual home as an integral part of their daily work, to ensure patient safety. This ability to adapt and make the best of each situation was emphasized as a crucial aspect of their professional responsibilities.

## 4. Discussion

This study aimed to explore how patient safety risks are experienced and managed in wound care in Swedish home healthcare settings from the healthcare professional perspective. The analysis showed that the participants primarily focused on underlying organizational preconditions that compromised patient safety in wound care, rather than on direct safety measures in clinical care. These patient safety risks were difficult to resolve. They related, in particular, to organizational challenges in terms of a lack of access to information, a lack of preventive measures, fragmented communication, a wide variety of conditions in patients’ homes, along with varying knowledge levels among the healthcare professionals. These constraints all relate to the WHO’s (2021) recommended patient safety framework, in which technologies, processes, procedures and environments are mentioned as organizational activities that are essential to achieving safe wound care.

Our main finding concerns organizational challenges that placed the healthcare professionals in situations that impeded their ability to provide safe care. Even if the healthcare professionals were cognizant of patient safety risks, they lacked the possibility to effectively address and manage them due to organizational circumstances that are beyond their control. The participants were aware of the limited possibilities, knowing that there were patient safety risks beyond their power to influence. Similarly, Lekman et al. (2023) addressed that patient safety risks were often given low priority because of structural deficiencies in the capacity for systematic risk prevention. Such circumstances have been reported to cause healthcare professionals to compensate for organizational shortcomings and provoke feelings of powerlessness in their situation (Silverglow et al., 2023). These well-known, wide-ranging effects of organizational management and structural factors on patient safety risks have largely been examined within a broader, more general patient safety perspective (Beer et al., 2014; Lindblad et al., 2018; Silverglow et al., 2023), elucidating several mechanisms through which such problems manifest.

In our current study, several organizational mechanisms critical to safe wound care were addressed by the participants. To begin with, discontinuity appeared to result in fragmentation of the wound care. This was associated with the presence of a diverse range of staff members, whose competencies varied substantially. Alvarez-Irusta et al. (2022) underscore that it is important to resolve problems of inadequate knowledge and lack of coordination to ensure continuity and promote safe wound healing. This includes improved collaboration with primary care physicians, which could benefit from appropriate and timely diagnosis and treatment (Ahmajärvi et al., 2022).

Another finding was discontinuities in communication. This was presented as another challenge related to the involvement of multiple actors, which resulted in the unavailability of essential information regarding the wound healing process and the measures taken to promote it. As posited by Lindh and Sahlqvist (2012), communication failure is a major underlying factor that contributes to adverse events, which underscores the pivotal role of efficacious communication in ensuring the provision of safe care. According to the participants, they were compelled to act only once problems had arisen, rather than take preventive measures. Such restrictions prevented them from working proactively. In congruence with our findings, Berland et al. (2012) reported that nurses in home healthcare settings took a reactive rather than proactive approach, resulting in treating patients after falling rather than preventing the fall in the first place. However, this was explained by a sense of unconcern and as something that was overshadowed by the emphasis on patient autonomy. This differs from our findings, in which the participants indicated that they desired additional organizational support to adopt a more proactive approach, a need that was unfulfilled. Overall, our findings demonstrate that unsafe wound care is a result of complex organizational mechanisms and not merely related to individual errors, which corresponds with the argument put forward by Cook et al. (2013). This further highlights the importance of addressing organizational preconditions to strengthen patient safety in wound care in home healthcare settings, thereby creating conditions that may also improve the management and prevention of a broader range of adverse events.

To summarize, the main finding showed that, because of limited freedom to operate, there remained a risk of unsafe care despite staff awareness of the risks and knowledge of prevention. This raises questions about situations in which injuries and suffering can be viewed as avoidable, that is, as adverse events. From a healthcare professional perspective, as the present findings suggest, such injuries and suffering could be viewed as involuntary (or forced) avoidable adverse events. In home healthcare settings, questions about the extent to which injuries caused by insufficient organizational preconditions are avoidable and how suffering can be prevented, therefore need to be addressed.

Overall, in the current study, the healthcare professionals appeared to be highly committed to making wound care safe. In light of Cook (2013), the unsafe care events that the healthcare professionals experienced can be understood as discontinuities in home healthcare settings that they both anticipated and identified as threats to patient safety, and which they acknowledged needed their attention. However, their efforts were not always enough. Thus, we agree with Lekman et al. (2023), who stated that overall structural changes in home healthcare may be needed to meet the existing requirements and advice of having management systems that take account of activities to promote patient safety (SOSFS, 2011:9; World Health Organisation, 2021). Essentially, such structures should establish accessible communication pathways for the exchange of information and be organized in a way that gives healthcare professionals scope for action that aligns with their professional capacity.

## 5. Strengths and limitations

In qualitative research, the criteria of credibility, dependability, confirmability, transferability, and authenticity are often used to assess the trustworthiness of the study (Graneheim & Lundman, 2004; Graneheim et al., 2017). The chosen methodology and the researchers’ extensive experience with it enabled an in-depth analysis, thereby strengthening the credibility of the findings. Dependability was enhanced through the use of an interview guide, which contributed to ensuring consistency in data collection among all participants. The confirmability of the study was enhanced by ensuring transparency throughout the analytical process, involving multiple researchers in the analysis, and the consistent use of quotations to track the basis of the interpretations. The use of direct quotations also strengthens the authenticity of the study, mirroring the perspectives and experiences of the participants and allowing readers to follow the interpretations. Transferability was strengthened by the detailed description of the study context, participant characteristics, and sampling strategy, which enable readers to assess the relevance of the findings to other settings.

Despite efforts to include a varied sample and strengthen the study’s transferability, several limitations should be acknowledged. The study was conducted in two small rural municipalities in southern Sweden, which may limit the transferability of the findings to larger urban areas or other healthcare systems. Nevertheless, the findings may still be relevant in settings where similar organizational and structural challenges shape the conditions for patient safety in home healthcare. Recruitment challenges resulted in an uneven distribution of participants between the two municipalities, as one was undergoing organizational restructuring. In addition, only two assistant nurses and one male participant contributed to the study, which restricted gender variation and the breadth of professional perspectives. A higher representation of assistant nurses could have provided a broader understanding of wound care practices, as registered nurses frequently described assistant nurses’ roles rather than elaborating on their own. These limitations may narrow the range of viewpoints represented; however, they also mirror the actual composition of Swedish home healthcare, where assistant nurses are less involved in wound care.

## 6. Conclusion

In conclusion, the findings indicate that healthcare professionals in home healthcare are committed to performing safe wound care. However, the organizational preconditions did not always allow them to act on identified patient safety risks, which resulted in imminent risks of adverse events and prolonged wound healing. The findings emphasize the importance of addressing organizational factors and conditions to strengthen patient safety in wound care. Thus, clinical implications need to be focused on establishing organizational preconditions that allow healthcare professionals to carry out their professional capabilities. Finally, as the findings show, the multifactorial patient safety measures addressed indicate that the voice of healthcare professionals must be acknowledged and acted upon in the home healthcare organization. Employing these changes may have the potential to enhance both patient outcomes and work satisfaction among healthcare professionals. Future research should explore and evaluate organizational and interprofessional interventions aimed at strengthening structural conditions and communication to support safe wound care in home healthcare.

## Data Availability

Data available on request owing to privacy/ethical restrictions.

## Generative artificial intelligence (AI)

Generative Artificial Intelligence (AI) tools (ChatGPT and DeepL) were used to assist with language improvement during the writing process. The final version of the manuscript was subsequently reviewed and revised by a professional language editor. The authors take full responsibility for the content of the manuscript.
